# Treatment-Induced BAFF Expression and B Cell Biology in Multiple Sclerosis

**DOI:** 10.3389/fimmu.2021.676619

**Published:** 2021-05-26

**Authors:** Ide Smets, Teresa Prezzemolo, Maya Imbrechts, Klara Mallants, Tania Mitera, Stéphanie Humblet-Baron, Bénédicte Dubois, Patrick Matthys, Adrian Liston, An Goris

**Affiliations:** ^1^ Department of Neurosciences, Laboratory for Neuroimmunology, KU Leuven, Leuven, Belgium; ^2^ Leuven Brain Institute, KU Leuven, Leuven, Belgium; ^3^ Department of Microbiology, Immunology and Transplantation, Laboratory for Adaptive Immunology, KU Leuven, Belgium; ^4^ VIB Center for Brain & Disease Research, Leuven, Belgium; ^5^ Department of Microbiology, Immunology and Transplantation, Rega Institute, Laboratory of Immunobiology, KU Leuven, Leuven, Belgium; ^6^ Department of Neurology, University Hospitals Leuven, Leuven, Belgium; ^7^ Laboratory of Lymphocyte Signalling and Development, The Babraham Institute, Cambridge, United Kingdom

**Keywords:** multiple sclerosis, B cells, B cell activating factor (BAFF), fingolimod, Interferon-β

## Abstract

Although fingolimod and interferon-β are two mechanistically different multiple sclerosis (MS) treatments, they both induce B cell activating factor (BAFF) and shift the B cell pool towards a regulatory phenotype. However, whether there is a shared mechanism between both treatments in how they influence the B cell compartment remains elusive. In this study, we collected a cross-sectional study population of 112 MS patients (41 untreated, 42 interferon-β, 29 fingolimod) and determined B cell subsets, cell-surface and RNA expression of BAFF-receptor (BAFF-R) and transmembrane activator and cyclophilin ligand interactor (TACI) as well as plasma and/or RNA levels of BAFF, BAFF splice forms and interleukin-10 (IL-10) and -35 (IL-35). We added an *in vitro* B cell culture with four stimulus conditions (Medium, CpG, BAFF and CpG+BAFF) for untreated and interferon-β treated patients including measurement of intracellular IL-10 levels. Our flow experiments showed that interferon-β and fingolimod induced BAFF protein and mRNA expression (P ≤ 3.15 x 10^-4^) without disproportional change in the antagonizing splice form. Protein BAFF correlated with an increase in transitional B cells (P = 5.70 x 10^-6^), decrease in switched B cells (P = 3.29 x 10^-4^), and reduction in B cell-surface BAFF-R expression (P = 2.70 x 10^-10^), both on TACI-positive and -negative cells. TACI and BAFF-R RNA levels remained unaltered. RNA, plasma and *in vitro* experiments demonstrated that BAFF was not associated with increased IL-10 and IL-35 levels. In conclusion, treatment-induced BAFF correlates with a shift towards transitional B cells which are enriched for cells with an immunoregulatory function. However, BAFF does not directly influence the expression of the immunoregulatory cytokines IL-10 and IL-35. Furthermore, the post-translational mechanism of BAFF-induced BAFF-R cell surface loss was TACI-independent. These observations put the failure of pharmaceutical anti-BAFF strategies in perspective and provide insights for targeted B cell therapies.

## Introduction

Interferon-β (IFN-β) and fingolimod are mechanistically very different multiple sclerosis (MS) treatments. Nonetheless, both converge in increasing B cell-activating factor (BAFF), newly formed and transitional B cells while decreasing memory B cells (1–5). BAFF is increasingly recognized as a key factor in B cell development, survival, immunoglobulin production and T cell stimulation (6). Transitional B cells are enriched for regulatory cells producing interleukin-10 (IL-10) (7) while memory B cells are more likely to have a pro-inflammatory function driving relapsing disease (8). A spontaneous BAFF increase has been observed in other autoimmune diseases and can correlate with disease progression (9, 10). Intriguingly, BAFF depletion is a therapeutic strategy in systemic lupus erythematosus (11) whereas it may exacerbate MS (12, 13). This highlights the importance of understanding the specific role of the BAFF-pathway in MS treatment. The BAFF-pathway is highly complex and it is unknown which mechanisms are responsible for the MS treatment-induced increase in BAFF levels. While BAFF itself is stimulatory, a BAFF splice form lacking an exon (deltaBAFF) can co-multimerize with full-length BAFF to oppose its function (14, 15). BAFF acts through different receptors, of which the two most important are BAFF-receptor (BAFF-R) and transmembrane activator and cyclophilin ligand interactor (TACI) (16, 17). Interactions between BAFF-receptors and CD40, a known B cell-related MS risk gene (18), have been reported (19, 20). To date, we do not fully understand how different MS treatments influence BAFF biology and direct the B cell compartment towards an immature phenotype. An improved understanding of their mode of action would put the failure of pharmaceutical anti-BAFF strategies in perspective and provide insights for targeted B cell therapies. In the present study, we evaluated in a cohort of untreated as well as IFN-β- or fingolimod-treated patients how treatments influence BAFF splice forms and receptors, and how these link to the cell subset shift within, and the regulatory function of, the B cell compartment.

## Materials and Methods

### Study Population

A study population of 112 unrelated patients of Caucasian descent fulfilling McDonald 2010 criteria for MS was included between May and November 2017 at the University Hospitals Leuven. We specifically selected untreated patients and patients treated with IFN-β or fingolimod. Overlap with our earlier work (1) is 14% (6/41 untreated), 21% (9/42 IFN-β) and 34% (10/29 fingolimod), respectively, where overlapping patients were sampled at an earlier time-point. The same treating physician (B.D.) collected clinical and demographic data during patient follow-up (**Table 1**). The study was approved by the Ethics committee of the University Hospitals Leuven (S60222).

**Table 1:** Study population.

Demographical/clinical characteristics	Untreated	Interferon-β	Fingolimod
Number of patients, N	41	42	29
Female/Male	31/10	24/18	16/13
Age (years), mean (± SD)	55.8 (± 12.5)	49.0 (± 11.8)	39.3 (± 10.6)
Age at onset (years), mean (± SD)	36.2 (± 11.5)	34.5 (± 11.4)	25.7 (± 9.0)
Disease course, BO/PP	32/9	42/0	29/0
Disease duration (years), mean (± SD)	20 (± 13)	15 (± 9.1)	14 (± 8.3)
MSSS, mean (± SD)	3.7 (± 2.6)	3.1 (± 2.8)	1.9 (± 1.7)
Treatment duration (years), mean (± SD)	–	7.5 (± 6)	4.5 (± 3.2)

(SD, Standard deviation; BO, bout onset; PP, primary progressive; MSSS, multiple sclerosis severity score).

### Cell Isolation and Storage

Heparinized blood was collected from patients to isolate peripheral blood mononuclear cells (PBMCs) using lymphocyte separation medium (Lymphoprep, Stemcell Technologies). PBMCs were frozen in 10% dimethyl sulfoxide (Sigma) in combination with foetal bovine serum and stored in liquid nitrogen.

### Immunophenotyping of B Cells Through Flow Cytometry

We processed PBMCs of the entire study population in four different batches. Frozen PBMCs were thawed, washed twice with PBS (Fisher Scientific) and stained with live/dead marker (Zombie Yellow™ Fixable Viability dye, eBioscience) and fluorochrome-conjugated antibodies against surface markers: anti-CD19 BV510, anti-CD24 BV421, anti-IgD APC-Cy7 (all BioLegend); anti-CD27 AF-700, anti-CD38 APC, anti-CD40 PE-Cy7, anti-BAFF-R FITC (all eBioscience); anti-CD86 PE-CF594, and anti-TACI BV650 (all BD Biosciences). A total of five B cell subpopulations could be measured using the following gating strategy as previously reported (18): total (CD19^+^), transitional (CD19^+^CD24^hi^CD38^hi^), naïve (CD19^+^CD27^-^), unswitched memory (CD19^+^CD27^+^IgD^+^) and switched memory B cells (CD19^+^CD27^+^IgD^-^). We measured expression of CD40, BAFF-R and TACI as percentage of positive cells for all five B cell subsets and as mean of fluorescence intensity (MFI) across positive cells for each cell type. The absolute cell counts of plasmablasts ﻿(CD19^+^CD24^-^CD38^hi^) were too low (< 100 cells) to reliably determine expression levels. Data were collected on BD Symphony flow cytometer (BD Biosciences). For data analysis, we used FlowJo (LLC, V10). **Supplementary Figure 1** shows representative FACS plots.

### Cell Cultures and Intracellular IL-10 Flow Cytometry

Cells were thawed and suspended in RPMI-1640 medium (HyClone) containing 10% FBS, penicillin (50 U/mL) and streptomycin (50 µg/mL). We enriched thawed PBMCs for B cells using the EasySep human B cell enrichment kit and brought 83,000 enriched B cells of six untreated and six IFN-β-treated patients in culture. Numbers of B cells were too low in PBMCs from fingolimod-treated patients in order to be included in this experiment. Cells were counted with a Bürker counting chamber. In both groups, we selected the patients with the highest percentages of transitional B cells within the B cell population as this is our main cell subset of interest. We cultured cells *in vitro* during 60 hours in 96-well plates. Cells were unstimulated or stimulated with human BAFF recombinant protein (50ng/mL, R&D systems) and/or CpG (1µg/mL, IDT). For intracellular staining, cells were stimulated for 4 hours in RPMI+10%FBS containing ionomycin (750ng/ml, Biotechne), PMA (100 ng/ml, Sigma) and brefeldin A (2µg/ml, Biotechne). After stimulation, we stained cells for surface markers [CD19-APCR700, CD24-BV711 (all BD Biosciences); CD27-APC-efluor780, CD38-PECy7, CD14-PerCP-Cy5.5, CD3-FITC (all eBioscience)] and viability [Zombie Aqua 516 (BioLegend)]. Cells were fixed and permeabilized according to the manufacturer’s protocol [(BD Biosciences) and stained intracellularly with antibodies against human IL-10 (IL-10-PE (BD Biosciences)]. Cells without BAFF/CpG stimulus were used as negative controls for cytokine staining. A total of four B cell populations were measured using the following gating strategy: total B (CD19^+^), transitional (CD19^+^CD24^hi^CD38^hi^), naïve (CD19^+^CD27^-^) and memory (CD19^+^CD27^+^) B cells. Flow cytometry was performed on a BD LSR Fortessa X20. Results were analysed with FlowJo (LLC, V10). **Supplementary Figure 2** shows representative FACS plots.

### Droplet Digital PCR

To quantify gene expression, we extracted and reverse transcribed RNA from total PBMCs using a high-capacity cDNA reverse transcription kit (Thermo Fisher). We determined the appropriate input concentration for low-abundant targets based on the lower limit of quantification (> 100 copies/well) and for high-abundant targets based on the average droplet saturation level (≤ 80%). RNA quantification on digital droplet PCR was conducted according to the manufacturer’s instructions with predesigned gene expression assays from Thermo Fisher. We used 15 ng cDNA for full-length BAFF (*TNFSF13B*, Hs00198106_m1), 50 ng cDNA for deltaBAFF (*TNFSF13B*, Hs04234382_m1), BAFF-R (*TNFRSF13C*, Hs00606874_g1), and TACI (*TNFRSF13B*, Hs00963364_m1), and 150 ng for *IL10* (Hs00961622_m1), IL12p35 (*IL12A*, Hs01073447_m1) and *EBI3* (Hs00194957_m1). Specificity of the BAFF-R gene expression assay, which may also amplify genomic DNA, for cDNA was verified by including non-transcribed RNA input. We measured the housekeeping genes *POLR2A* (Hs00172187_m1, 15 ng input), *IPO8* (Hs00183533_m1, 50 ng input), *MRPL19* (Hs00608519_m1, 50 ng input) and *HPRT1* (Hs99999909_m1, 50 ng input). We recalculated all measured gene expression levels to reflect the amount expressed using an input concentration of 50 ng. We normalized the target gene expression by the average expression of four housekeeping genes.

### Cytokine Quantification

In patients, we measured circulating plasma levels of BAFF using a human BAFF Quantikine ELISA (R&D Systems) and plasma IL-10 by electrochemiluminescence immunoassay using the Meso Scale Discovery plates. We performed all measurements on the entire study population on two 96-well plates including a duplicate eight-point standard curve.

### Statistical Analysis

In the study population of 112 individuals, we omitted from the analysis missing data points or outlier measurements deviating more than five standard deviations from the mean at immune, protein, RNA or DNA level. Sample size for each analysis is included in the figure legends. Using R v3.6.1, we performed a linear regression of the immunological parameters obtained with flow cytometry in function of treatment status, age and gender. If there was suspicion that statistical significance could be driven by extreme datapoints, a sensitivity analysis was performed by repeating the linear regression without the extreme data points. For correlation analysis, a linear regression was performed between a dependent and independent variable with age, gender and treatment as a covariate. To test for differences between the stimulation conditions, we used a dependent-samples Sign-test. To test for differences between untreated and IFN-β treated patients in the stimulation experiment, we used a Wilcoxon test. We applied a Bonferroni correction factor for multiple testing of 30 cellular variables (based on both % positive cells and MFI tested for three cell surface molecules in five B cell populations) generating a corrected significance threshold P value of 0.0017. This multiple testing correction is actually highly conservative given the extensive correlation between many of the assessed variables on flow cytometry. In our follow-up experiments (RNA, cytokine, correlations and cell culture experiments), we applied a nominal significance threshold (P = 0.05).

## Results

We collected PBMCs from a cross-sectional study population of 112 MS patients, of which 41 were untreated and 42 and 29 were treated with IFN-β or fingolimod, respectively (**Table 1**). Fingolimod treated patients were on average younger than IFN-β and untreated patients and had a shorter disease and treatment duration.

### BAFF Increase Induced by MS Treatments Modulates the B Cell Compartment to an Early B Cell State

**Figure 1:**
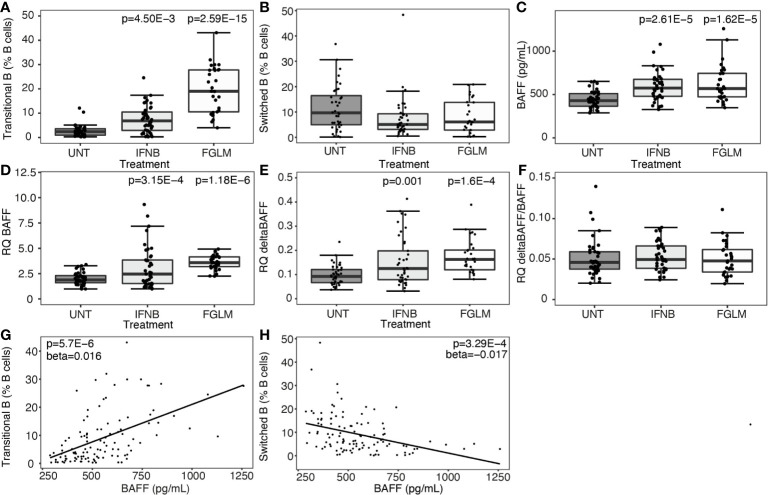
Expression of BAFF and BAFF spliceforms according to treatment status. Association of treatment status (UNT = untreated, IFNB = interferon-β, FGLM = fingolimod) with **(A)** transitional B cells (NUNT = 41, NIFNB = 42, NFGLM = 29), **(B)** switched memory B cells (NUNT = 40, NIFNB = 42, NFGLM = 29), **(C)** BAFF protein levels (NUNT = 41, NIFNB = 42, NFGLM = 29), **(D)** relative quantity (RQ) of full-length BAFF (NUNT = 41, NIFNB = 42, NFGLM = 29), **(E)** relative quantity (RQ) of deltaBAFF (NUNT = 41, NIFNB = 42, NFGLM = 29), **(F)** the ratio of deltaBAFF versus full-length BAFF (NUNT = 41, NIFNB = 41, NFGLM = 29). Correlation of BAFF protein levels with **(G)** transitional B cells and **(H)** switched memory B cells. P-values ≤ 0.05 are depicted and were calculated from linear regression of the immunological or expression variable in function of treatment status (reference = UNT) with age and gender as a covariate. P values for the correlation of protein BAFF with immune subsets was calculated with a linear regression (reference = UNT) with treatment, age and gender as covariates. Box-whisker plots represent median, quartiles and 1.5 x IQR.

At cellular level, we observed a strong step-wise increase in the percentage of transitional B cells from IFN-β (P = 4.50 x 10^-3^) to fingolimod (P = 2.59 x 10^-15^) compared to untreated MS patients (**Figure 1A**). We did not observe a significant decrease in switched memory B cells (**Figure 1B**). Soluble BAFF protein levels as well as BAFF mRNA expression levels were increased in IFN-β and fingolimod (P ≤ 3.15 x 10^-4^) (**Figures 1C, D**). The increase in full-length BAFF mRNA was mirrored by a proportional rise in deltaBAFF mRNA lacking exon 3 (ratio: P ≥ 0.61) (**Figures 1E, F**). To understand how cellular and cytokine alterations interact, we looked at correlations between both levels. Plasma BAFF levels correlated with an increase in transitional B cells (P = 5.70 x 10^-6^) (**Figure 1G**) and a decrease in switched B cells (P = 3.29 x 10^-4^) (**Figure 1H**). The positive correlation was specific for transitional B cells and no association surviving multiple testing was seen for naïve B cells (P = 0.0029).

### Treatment-Induced BAFF Is Associated With BAFF-R Loss Independent of TACI

**Figure 2:**
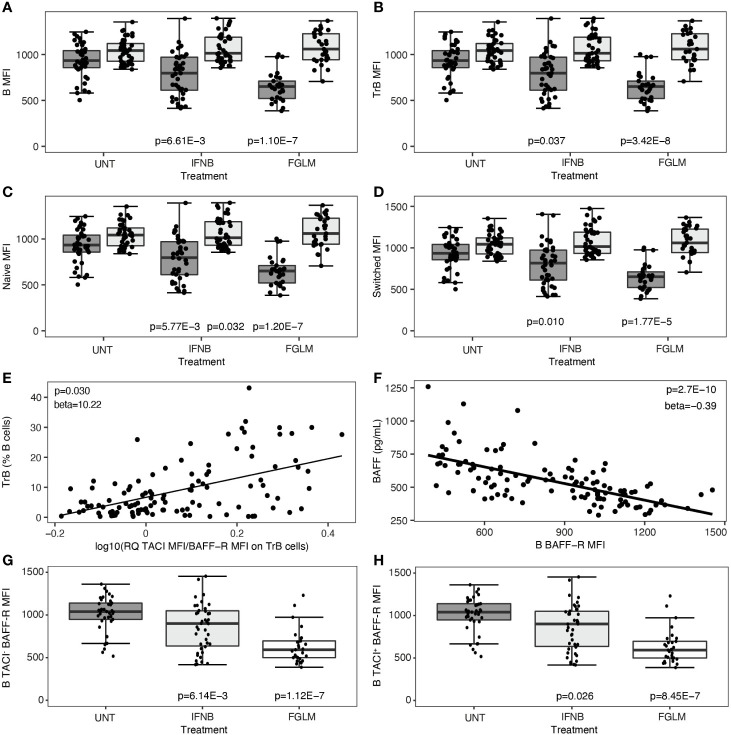
B cell surface expression of BAFF-R and TACI according to treatment status. Association of treatment status (UNT = untreated, IFNB = interferon- β, FGLM = fingolimod) with mean fluorescence intensity (MFI) of BAFF-R (dark grey) and TACI (light grey) on **(A)** total B, **(B)** transitional B, **(C)** naïve B and **(D)** switched memory B cells (NUNT = 41, NIFNB = 42, NFGLM = 29). **(E)** Correlation of transitional B cells with the ratio of the mean fluorescence intensity (MFI) of TACI on BAFF-R on transitional B cells. **(F)** Correlation between BAFF-R expression level on total B cells and BAFF plasma concentration. Association of treatment status with mean fluorescence intensity (MFI) of BAFF-R on **(G)** TACI- total B and **(H)** TACI+ total B cells (NUNT = 41, NIFNB = 42, NFGLM = 29). P values ≤ 0.05 are depicted. For plot **(A–D, G, H)**: P values were calculated with a linear regression of the immunological or expression variable in function of treatment status with age and gender as a covariate. For plot **(E, F)**: P values were calculated with a linear regression of BAFF plasma level/TrB cells in function of receptor expression levels with age, gender and treatment status as a covariate. We measured expression of BAFF-R and TACI as mean of fluorescence intensity (MFI) across positive cells for each cell type. Box-whisker plots represent median, quartiles and 1.5 x IQR.

Subsequently, we investigated changes in the two most important BAFF-receptors, BAFF-R and TACI, after treatment. This was quantified both as percentage of positive cells and as mean cell surface expression levels (MFI). In fingolimod-treated patients, the decrease in BAFF-R expression was highly significant in all evaluated B cell subsets (1.77 x 10^-5^ ≤ P ≤ 3.42 x 10^-8^) and most manifest in terms of effect size on transitional B cells (**Figures 2A–D**). This observation was paralleled by a significant decline in the percentage of BAFF-R^+^ B cells and subsets (5.44 x 10^-4^ ≤ P ≤ 1.54 x 10^-8^) (**Supplementary Figure 1**). In IFN-β-treated patients, likewise a consistent decrease in BAFF-R cell-surface expression levels was seen (0.010 ≤ P ≤ 5.77 x 10^−3^) (**Figures 2A–D**), although the same step-wise effect across treatments as for BAFF levels above meant this did not survive conservative Bonferroni-correction. The levels of TACI cell-surface expression and the percentage of TACI^+^ cells remained remarkably constant in all B cell subsets in both treatments (**Figures 2A–D**, **Supplementary Figure 3**). These changes resulted in an increased ratio of TACI on BAFF-R cell surface expression in all B cell subsets. In particular for transitional B cells, this increased ratio correlated modestly with the frequency of transitional B cells (P = 0.030) (**Figure 2E**). The steep reduction of BAFF-R expression on total B cells was inversely proportional to BAFF plasma levels (P = 2.70 x 10^-10^) (**Figure 2F**). We observed no significant differences surviving multiple testing correction in CD40 expression level or percentage CD40^+^ B cells in treated versus untreated patients (**Supplementary Figure 4**). In contrast to cell surface protein levels, we could not observe any treatment-induced differences in TACI/BAFF-R ratio when assessing mRNA expression levels of individual receptors (P ≥ 0.058) (data not shown), suggesting a post-translational mechanism such as shedding of BAFF-R. As BAFF-R shedding may be TACI-dependent, we distinguished between TACI-positive and TACI-negative cells for all B cell subsets. The reduction of BAFF-R expression on the cell surface across treatments was equally pronounced in TACI^-^ versus TACI^+^ total B cells (**Figures 2G, H**), and all B cell subsets (**Supplementary Figure 5**).

### Treatment-Induced BAFF Changes Do Not Stimulate Immunoregulatory Cytokines in B Cells

**Figure 3:**
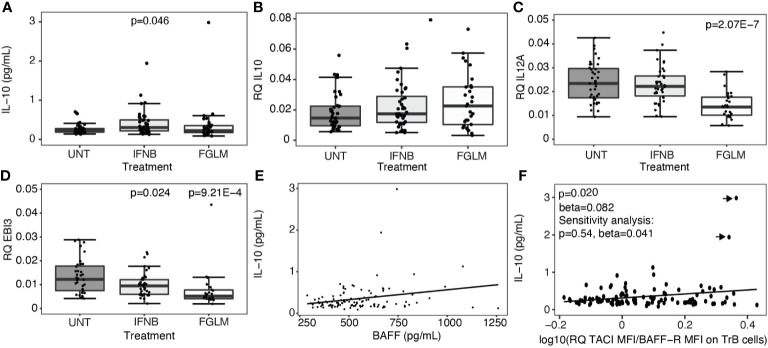
Expression of regulatory cytokines according to treatment status. Association of treatment status (UNT = untreated, IFNB = interferon- β, FGLM = fingolimod) with **(A)** IL-10 plasma cytokine levels (NUNT = 40, NIFNB = 42, NFGLM = 29) and **(B)** the relative quantity (RQ) of IL10 (NUNT = 41, NIFNB = 40, NFGLM = 29), **(C)** RQ of IL12p35 (NUNT = 39, NIFNB = 38, NFGLM = 27) and **(D)** RQ of EBI3 (NUNT = 36, NIFNB = 38, NFGLM = 26). **(E)** Correlation of IL-10 with BAFF plasma levels. **(F)** Correlation of IL-10 plasma levels with the ratio of the mean fluorescence intensity (MFI) of TACI on BAFF-R on transitional B cells. P-values ≤ 0.05 are depicted. **(A-D)**: P values were calculated from linear regression of the gene expression variable in function of treatment status (reference = UNT) with age and gender as a covariate. **(E, F)**: P values were calculated with a linear regression of IL-10 levels in function BAFF/receptor expression levels with age, gender and treatment status as a covariate. Data points omitted in the sensitivity analysis are indicated with arrows. Box-whisker plots represent median, quartiles and 1.5 x IQR.

Regulatory B cells, producing the anti-inflammatory cytokines interleukin-10 (IL-10) and interleukin-35 (IL-35), are enriched amongst transitional B cells. We measured expression of IL-10 plasma protein levels and IL-10 as well as IL-35 subunits, IL-12p35 and EBI3, at the RNA level in total PBMCs. A significant increase in plasma IL-10 was limited to IFN-β-treated patients (P = 0.046) (**Figure 3A**). At the RNA level, no changes were seen for IL-10 (**Figure 3B**), and treatment even induced a decrease in expression of IL-35 (**Figures 3C, D**). In contrast to the correlations seen for transitional B cells, neither BAFF (**Figure 3E**) nor the ratio of TACI over BAFF-R (**Figure 3F**) protein levels correlated with plasma IL-10 levels.

**Figure 4:**
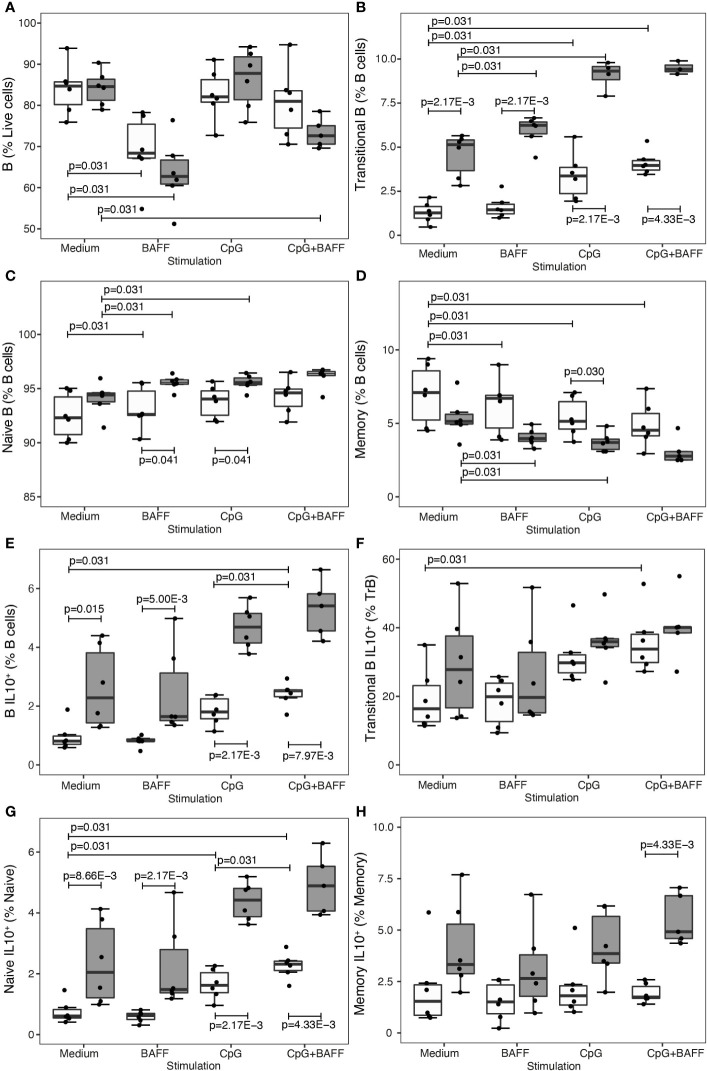
BAFF enhances B cell subset shift, but not IL-10 production, in treated patients. Association of treatment status (UNT = untreated, visualized in white; IFNB = interferon-β, visualized in dark grey) and stimulation condition (medium, BAFF, CpG or BAFF + CpG) with **(A)** B cells (NMedium= 6 UNT, 6 IFNB; NBAFF= 6 UNT, 6 IFNB; NCpG= 6 UNT, 6 IFNB; NCpG+BAFF= 6 UNT, 6 IFNB), **(B)** transitional B cells (NMedium= 6 UNT, 6 IFNB; NBAFF= 6 UNT, 6 IFNB; NCpG= 6 UNT, 6 IFNB; NCpG+BAFF= 6UNT, 5 IFNB), **(C)** naïve B cells (NMedium= 6 UNT, 6 IFNB; NBAFF= 6 UNT, 6 IFNB; NCpG= 6 UNT, 6 IFNB; NCpG+BAFF= 6 UNT, 5 IFNB), **(D)** memory CD27+ B cells (NMedium= 6 UNT, 6 IFNB; NBAFF= 6 UNT, 6 IFNB; NCpG= 6 UNT, 6 IFNB; NCpG+BAFF= 6 UNT, 5 IFNB), **(E)** IL10+ B cells (NMedium= 6 UNT, 6 IFNB; NBAFF= 6 UNT, 6 IFNB; NCpG= 6 UNT, 6 IFNB; NCpG+BAFF= 6 UNT, 5 IFNB), **(F)** IL10+ transitional B cells (NMedium= 6 UNT, 6 IFNB; NBAFF= 6 UNT, 6 IFNB; NCpG= 6 UNT, 6 IFNB; NCpG+BAFF= 6 UNT, 5 IFNB), **(G)** IL10+ naïve B cells (NMedium= 6 UNT, 6 IFNB; NBAFF= 6 UNT, 6 IFNB; NCpG= 6 UNT, 6 IFNB; NCpG+BAFF= 6 UNT, 5 IFNB) and **(H)** IL10+ memory CD27+ B cells (NMedium= 6 UNT, 6 IFNB; NBAFF= 6 UNT, 6 IFNB; NCpG= 6 UNT, 6 IFNB; NCpG+BAFF= 6 UNT, 5 IFNB). To test for differences between untreated and IFNB treated patients, we used a Wilcoxon test. To test for differences between medium and other stimulation conditions or CpG and CpG + BAFF within the IFNB treated and untreated patients, we used a Sign-test. P values ≤ 0.05 are depicted. Box-whisker plots represent median, quartiles and 1.5 x IQR.

As IL-10 plasma or RNA assays have limited sensitivity *ex vivo*, we additionally included intracellular IL-10 flow cytometry in an *in vitro* experiment where B cells of untreated and IFN-β-treated patients were cultured for 60 hours in medium or medium with B cell stimuli CpG and/or BAFF (**Figures 4A–H**). In B cells from IFNB-treated patients, both transitional B cells (P = 2.17 x 10^-3^) (**Figure 4B**) and IL-10 producing B cells (P ≤ 0.015) (**Figure 4E**) were proportionally increased compared to untreated patients. CpG induced a shift towards more transitional B cells and less memory B cells in the untreated as well as IFN-β condition (**Figures 4B, D**). Moreover, CpG alone or in combination with BAFF induced IL-10 producing B cells, particularly in the naïve subset, in untreated MS patients (**Figures 4F, G**). Under the influence of BAFF stimulation, we found that total live B cells decreased after 60 hours of culture (P = 0.031) (**Figure 4A**). Regarding the B cell subsets, BAFF induced a modest rise in the fraction of transitional and naïve B cells whereas the relative amount of memory B cells was reduced (**Figures 4B–D**). In contrast to CpG, BAFF alone did not exert an influence on the intracellular IL-10 production capacity in any of the B cell subsets (**Figures 4E–H**).

## Discussion

IFN-β and fingolimod converge in inducing a BAFF increase at the RNA and protein level that is correlated with a sharp increase in the numbers of transitional B cells. DeltaBAFF is the most established alternative splice form of BAFF missing exon 3 and as a consequence lacking 57 nucleotides while including an additional glycosylation site. This splice form is highly conserved between mice and humans. In cell culture and animal models, deltaBAFF can exert an antagonizing function on full-length BAFF and alterations in the ratio of the two splice forms may play a role in limiting BAFF bioavailability (14, 15). In our *ex vivo* human approach, however, we found that upon MS treatment the BAFF alternative splice form was proportionately upregulated compared to the full-length form and the ratio between the two was unchanged.

Treatment with IFN-β and fingolimod significantly altered the ratio between B cell surface expression of the two most important BAFF receptors, BAFF-R and TACI. Under the influence of treatment, there was a BAFF-induced decrease of BAFF-R cell surface expression on all B cell subsets. On the RNA level, BAFF-R expression remained unchanged implying a role for BAFF-induced post-translational processes. Feedback mechanisms adjusting BAFF and BAFF-R levels to the requirements of BAFF-dependent B cell subsets have previously been observed in mice and primary immunodeficiency patients (21, 22). For TACI, on the contrary, our study showed that RNA and cell surface expression was steady and unaltered in the light of treatment, extending on previous observations of soluble TACI dynamics in fingolimod treated subjects (5). This contradicts earlier results in mice demonstrating an expansion of TACI^+^ transitional B cells in response to BAFF (23). Our data on BAFF-R changes upon treatment were not seen in *in vitro* experiments (5), which suggests this type of analysis may not fully reflect the mechanism of action of fingolimod *in vivo*. Moreover, our data were only partially compatible with the recently reported mechanism in which BAFF-R undergoes ligand-induced shedding (24). In that study, BAFF-R shedding was reported to be TACI-dependent as shedding was only observed in human EBV cell lines or mouse cell lines co-expressing BAFF-R and TACI. In contrast, in our *ex vivo* samples BAFF-induced BAFF-R loss occurred both in TACI^+^ and in TACI^-^ B cells, implying that TACI-independent mechanisms must be involved such as for example shedding or ligand-dependent internalisation and degradation, which require further investigation. These differences between *in vitro* and *ex vivo* data may be related to different members of the A Disintegrin And Metalloproteinase domain-containing protein (ADAM) family involved in cleaving the receptor from the cell surface (24). Notably, ADAM17 has been implicated in BAFF-R shedding in B cell lines, where TACI-dependence was seen, whereas ADAM10 cleaves BAFF-R in primary B cells, such as in our patient cohort where we do not observe BAFF-R shedding being dependent on TACI. Activation of the two receptors by BAFF could lead to opposing effects on B cells, and the increase of TACI over BAFF-R may result in an inhibitory signal, leading to less proliferation and to apoptosis (19, 25). On the other hand, different B cell subsets have different affinities for BAFF-driven survival signals. B cell maturation arrests in the transitional stage when BAFF-R is deficient (26, 27) whereas the survival of memory B cells is – in mice at least – largely BAFF independent (28, 29). In our data post-treatment, the higher ratio of TACI over BAFF-R specifically on transitional B cells was correlated with an increased proportion of transitional B cells within the B cell pool.

Transitional B cells are enriched in IL-10 producing cells. Their relative abundance contributes to a more regulatory immune state (7, 30). However, our *ex vivo* and *in vitro* data did not underpin a negative or positive direct effect of BAFF or BAFF-R shift on IL-10 production. As a positive control, we replicated the previously described effects of IFN-β and CpG on B cell IL-10 production (31, 32), but this is independent of and not augmented by a BAFF stimulus under treatment. This contrasts with animal data and human data from lymphoproliferative or other autoimmune diseases showing the involvement of BAFF in inducing a regulatory B cell phenotype through TACI signalling (33, 34). In particular, human and mice monoclonal chronic lymphocytic leukaemia B cells increased intracellular IL-10 production upon stimulation with BAFF and CpG *versus* either condition alone, and this was reduced by blocking TACI (34). In human healthy donors, on the other hand, at most a very modest increase in IL-10 production by BAFF and CpG *versus* CpG and no effect of BAFF only was seen. This is in line with our data on MS patients. A similar observation was done for IL-35, another cytokine reported to have a B cell regulatory function in mice (35, 36). MS treatments inducing the BAFF pathway did not increase, indeed, they even decreased, expression of IL-35 and its subunits. Although an *in vitro* effect of IFN-β on CD40 has previously been reported (31), our *ex vivo* data do not demonstrate a significant shared effect of IFN-β and fingolimod on CD40 B cell surface expression. Altogether, our findings suggest that changes in the BAFF pathway induced upon MS treatment contribute to a shift in B cell subset composition towards transitional B cells but do not upregulate B cell regulatory cytokines. Other pathways may be involved in IL-10 production in B cells. In MS patients with helminth infections, there is a compensatory abundance of IL-10-producing B cells through a mechanism that is largely dependent on the Inducible T cell costimulator ligand (ICOSLG)-pathway and not on the CD40, CD80 or CD86 pathways (37). Further investigation is required to understand whether the same mechanism leads to IL-10 upregulation in current pharmacological MS treatments.

Our data shed further light on current concepts regarding BAFF in autoimmune disease. Blocking BAFF using a range of monoclonal antibodies reduces immunoglobulin G and auto-antibody titres (11, 12), which may explain its registered use in antibody-positive systemic lupus erythematosus (SLE) where it reduces organ damage and disease flares (11). Clinical trials blocking BAFF in MS warranted by animal models also decreased antibody titres (12) but failed in terms of treatment outcome in MS, where B cell subset shifts appear more important (12, 13, 38). Immunologically, BAFF depletion reduces transitional and naïve B cells while increasing memory B cells. This is the exact opposite of the B cell dynamics under the influence of established MS treatments, including the two treatments in our study (39). Based on our work, we can now hypothesize that BAFF induces changes in the ratio of BAFF receptors which in turn contribute to B cell subset shifts. Observations on different disease mechanisms of the BAFF pathway underlying the amalgam of autoimmune diseases are in line with the CD40 dichotomy. The same *CD40* single nucleotide polymorphism is shared across autoimmune diseases but with opposite effects in antibody-related diseases such as SLE *versus* MS, where the role of shifts in B cell subsets is highlighted again (18). Transitional B cells are precursors for mature B cells. They are enriched for IL-10 producing cells, and any shift towards transitional B cells would increase these cells proportionally. However, all B cell subsets, including naïve and memory subsets, are able to produce IL-10 (7, 32). In addition, a reduction in memory B cells decreases the pro-inflammatory state of the immune system (8). Our data indicate that IL-10 induction in MS does not depend on the BAFF-BAFF-R pathway. Hence, therapeutic strategies to foster the function of IL-10 need to be explored independently of the B cell subset shift induced by BAFF.

Our study was based on a routine clinical setting in a tertiary outpatient clinic. While we corrected for heterogeneity in age and sex, additional variables affecting treatment decisions, patient compliance, and treatment response are potential confounders. Fingolimod induces, apart from a B cell subset shift, also a strong decrease in T and B cell numbers in the peripheral blood. This limits the feasibility of experiments starting from sorted B cells, as for our *in vitro* data. However, we focus on mechanisms concerning the BAFF-pathway that have been demonstrated as a convergence between treatments. For this purpose, we were able to collect blood samples from a large study population of MS patients. ﻿Whereas we were only able to assess changes in the peripheral blood, and not the target tissue, there is active exchange between the CNS and peripheral blood (40, 41), and changes in the peripheral blood have previously been shown to be able to capture genetic variation important for MS (18). Moreover, immunomodulatory treatments are not administered directly into the CNS, and thus the likely location of activity is in the periphery, where our analysis took place. In addition, we realize that our *ex vivo* human data might differ from previously reported *in vivo* animal data and do not allow to make a distinction between association and causation. However, we believe that these *ex vivo* human data are highly important in a context where clinical trials with treatments that were highly promising in and mechanistically underscored by animal data have failed in human patients, and where the reason for their failure remains currently unexplained (12, 13).

In summary, MS treatments induce signalling through the BAFF-BAFF-R pathway which redirects the B cell compartment towards transitional B cells without change in IL-10 levels. Similarly, there was no role for IL-35, CD40 and TACI. Our observations regarding the BAFF receptor dynamics further highlight the disparity between data collected from animal models or other immune diseases *versus* human MS. Therefore, careful scrutiny of the human B cell compartment in MS patients is necessary to guide future B cell-targeted therapies.

## Data Availability Statement

The raw data are available at KU Leuven and will be shared on request from any qualified investigator pending Institutional Review Board approval and accordance with EU General Data Protection Regulation.

## Ethics Statement

The studies involving human participants were reviewed and approved by Ethics committee of the University Hospitals Leuven (S60222). The patients/participants provided their written informed consent to participate in this study.

## Author Contributions

IS, PM, BD, AL and AG contributed to conception and design of the study. IS, TP, KM, MI, TM performed lab experiments, and IS performed the statistical analysis. IS wrote the first draft of the manuscript. All authors contributed to the article and approved the submitted version.

## Funding

IS holds a European Committee for Treatment and Research in Multiple Sclerosis Clinical Fellowship. BD is a Clinical Investigator of the Research Foundation Flanders (FWO). This study was supported by Research Fund KU Leuven (C24/16/045), Research Foundation Flanders (FWO G.0734.15N), Belgian Charcot Foundation, and Queen Elisabeth Medical Foundation (Research Project and Prize Viscountess Valine de Spoelberch).

## Conflict of Interest

BD has received consulting fees and/or funding from Biogen Idec, BMS, Sanofi-Aventis and Teva. AG and BD have received consulting fees, travel funding and/or research funding from Roche, Novartis and Merck.

The remaining authors declare that the research was conducted in the absence of any commercial or financial relationships that could be construed as a potential conflict of interest.

## Abbreviations

ADAM, A disintegrin and metalloproteinase domain-containing protein; BAFF, B-cell activating factor; BAFF-R, B-cell activating factor receptor; ICOSLG, Inducible T cell costimulator ligand; IL-10, Interleukin 10; IFN-β, Interferon-β; FGLM, Fingolimod; MFI, Mean fluorescent intensity; MS, Multiple sclerosis; PBMCs, Peripheral blood mononuclear cells; RQ, Relative quantity; TACI, Transmembrane activator and cyclophilin ligand interactor; UNT, Untreated.
