# Supplementary material for: Treatment-Induced BAFF Expression and B Cell Biology in Multiple Sclerosis
Source: Front Immunol. 2021 May 26;12:676619. doi: 10.3389/fimmu.2021.676619 (PMC8187869; doi:10.3389/fimmu.2021.676619)

**Supplementary material**

Supplementary Figure 1: B cell flow cytometry gating strategy for one representative sample. **A.** Single cell gated on forward scatter height vs. area, **B.** Lymphocyte gate selected on side scatter (SSC-A) vs. forward scatter (FSC-A). **C.** Live cells selected based on absence of Zombie Yellow expression, **D.** CD19^+^ gate selected on live cells. **E.** BAFF-R^+^, **F.** TACI^+^, **G.** CD40^+^ gate selected on B cells. **H.** Transitional B cell (TrB) and plasmablast (PB) gate selected on CD38 vs. CD24 of B cells. **I.** BAFF-R^+^, **J.** TACI^+^, **K.** CD40^+^ gate selected on Transitional B cells. **L.** Naive B cell gate selected on CD27 of B cells, unswitched and switched memory B cells selected on CD27 and IgD of B cells and **M. P. S.** BAFF-R^+^, **N. Q. T.** TACI^+^, **O. R. U.** CD40^+^ gate selected on respectively naive, unswitched memory and switched memory B cells.


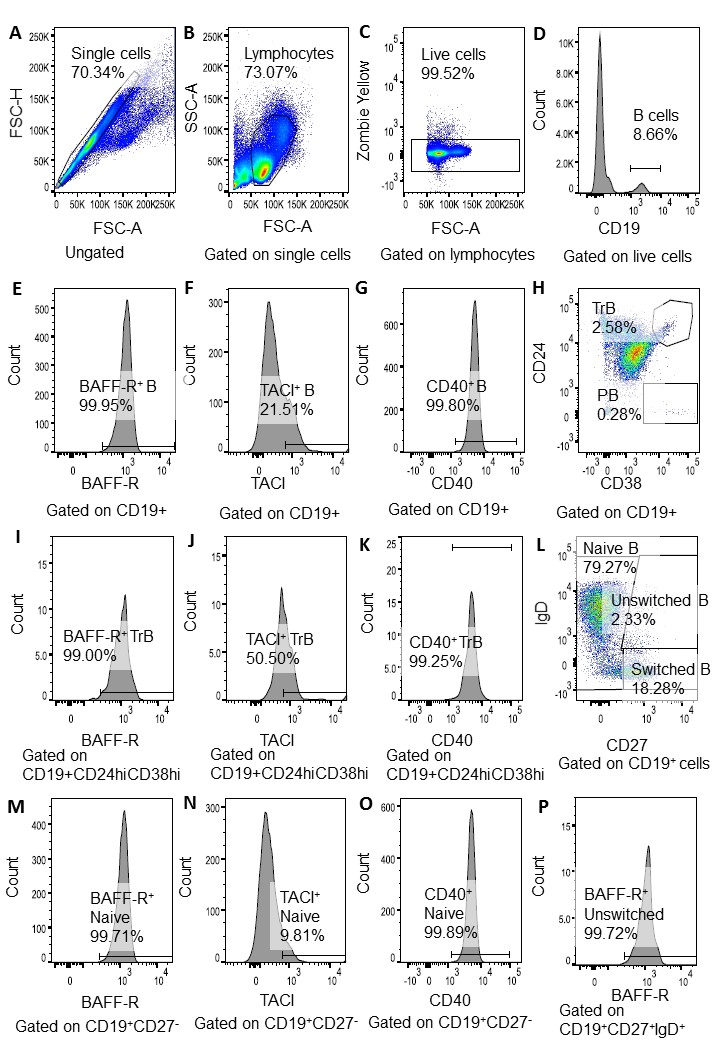


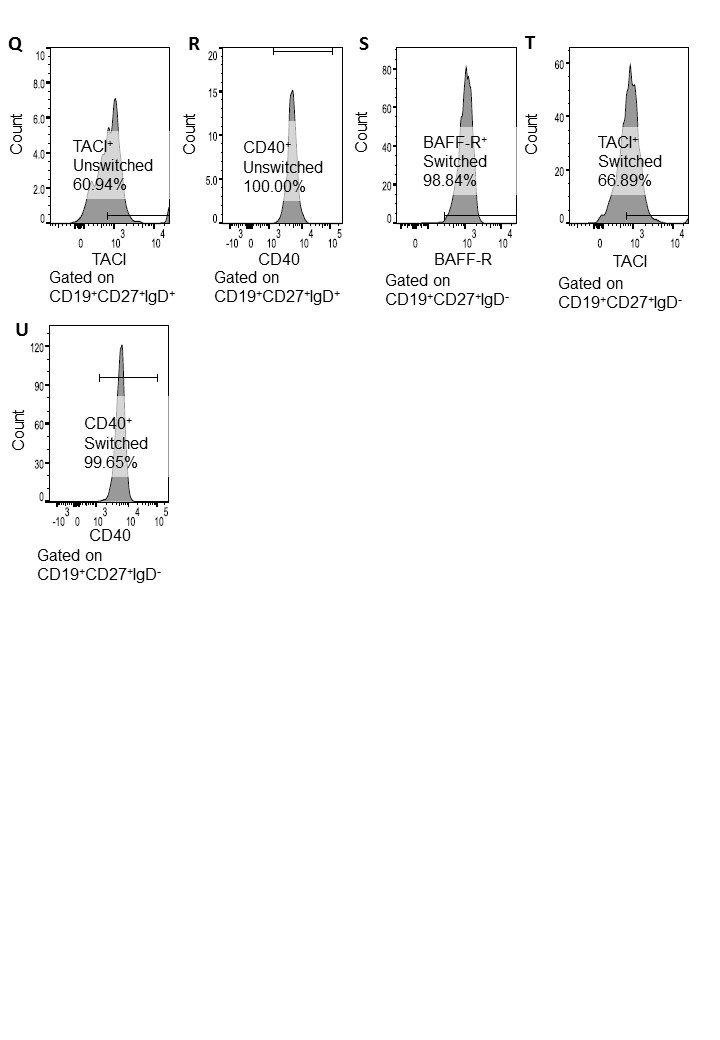


**
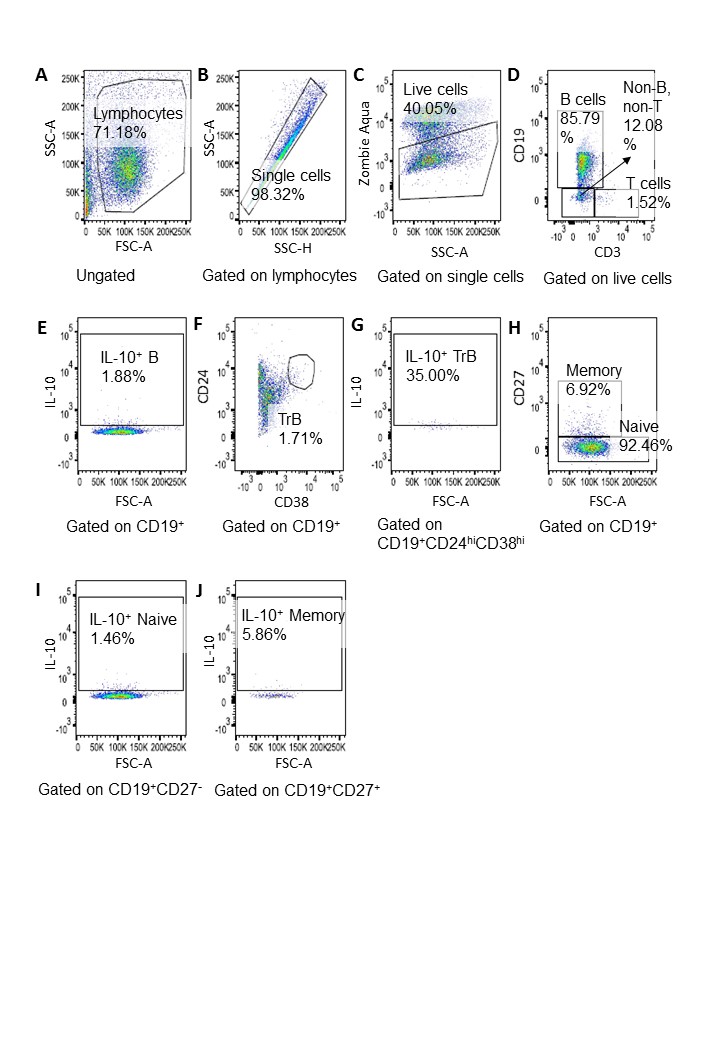
Supplementary Figure 2: Intracellular IL-10/B cell flow cytometry gating strategy for one representative sample. A**. Lymphocyte gate selected on side scatter (SSC-A) vs. forward scatter (FSC-A). **B.** Single cell gated on forward scatter height vs. area **C.** Live cells selected based on the absence of Zombie Aqua expression, **D.** CD19^+^, CD3^+^ and CD19^-^CD3^-^ gate selected on live cells. **E.** IL-10^+^ gate selected on B cells. **F.** Transitional B cell (TrB) gate selected on CD38 vs. CD24 of B cells. **G.** IL-10^+^ gate selected on Transitional B cells. **H.** Naive and memory B cell gate selected on CD27 of B cells. **I. J.** IL-10^+^ gate selected on respectively naive and memory B cells.

Supplementary Figure 3: Percentage of B cells expressing BAFF-R and TACI according to treatment status. Association of treatment status (UNT = untreated, IFNB = interferon-β, FGLM = fingolimod) with percentage of BAFF-R (dark grey) and TACI (light grey) positive cells across **A.** total B, **B.** transitional B, **C.** naive B, **D.** switched memory B cells. P values were calculated with a linear regression of the immunological variable in function of treatment status (reference = UNT) with age and gender as a covariate. Only P values ≤ 0.05 are depicted. Box-whisker plots represent median, quartiles and 1.5 x IQR.


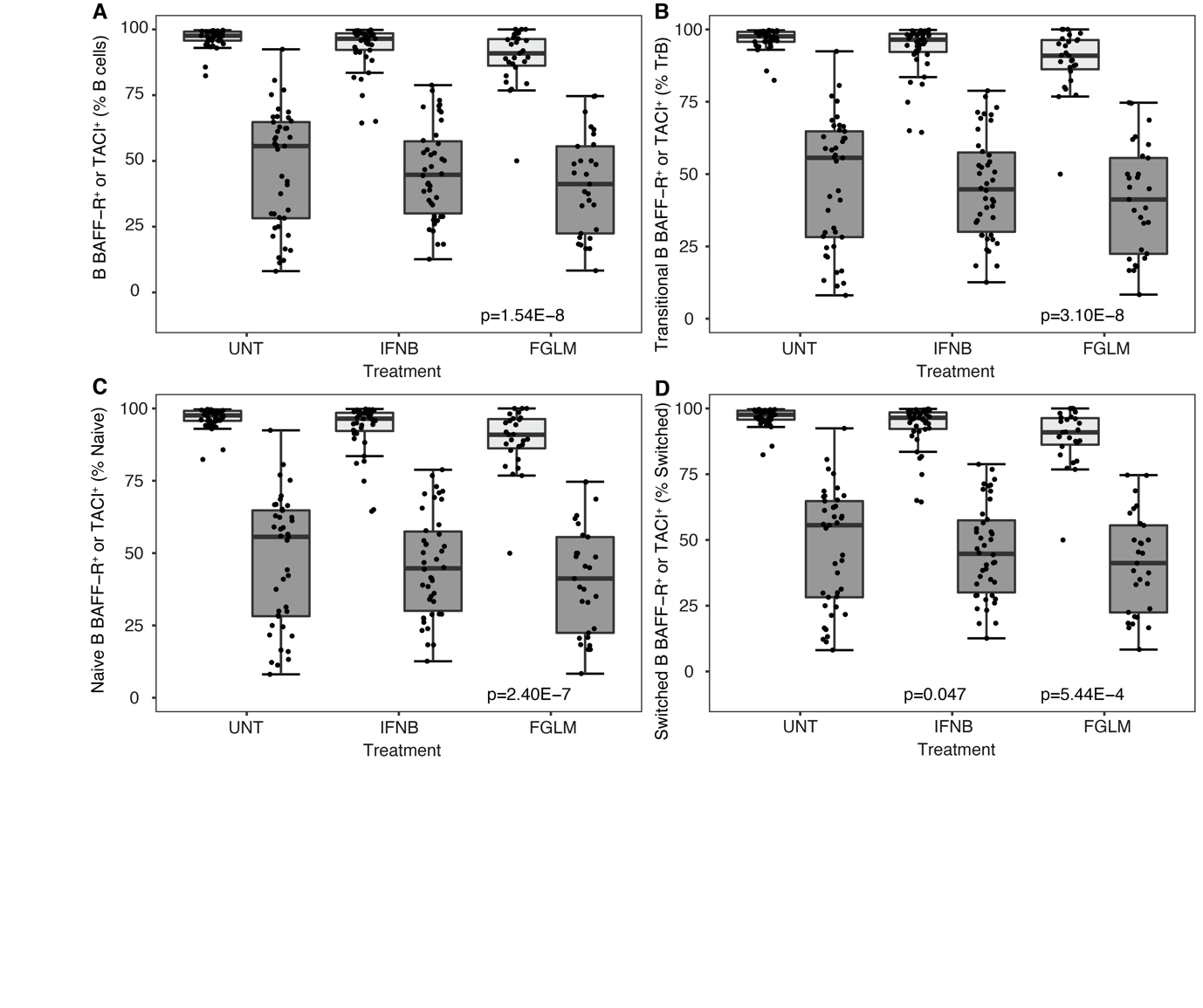


**Supplementary Figure 4: Percentage of B cells expressing CD40 according to treatment status.** Association of treatment status (UNT = untreated, IFNB = interferon- β, FGLM = fingolimod) with percentage of cells expressing CD40 in **A.** total B, **B.** transitional B, **C.** naive B and **D.** switched memory B. Association of treatment status with mean fluorescence intensity (MFI) of CD40 on **E.** total B, **F.** transitional B, **G.** naive B and **H.** switched memory B cells. P values were calculated with a linear regression of the immunological variable in function of treatment status (references = UNT) with age and gender as a covariate. We measured expression of CD40 as mean of fluorescence intensity (MFI) across positive cells for each cell type. Only P values ≤ 0.05 are depicted and the y-axes range from 75 to 100%. Box-whisker plots represent median, quartiles and 1.5 x IQR.


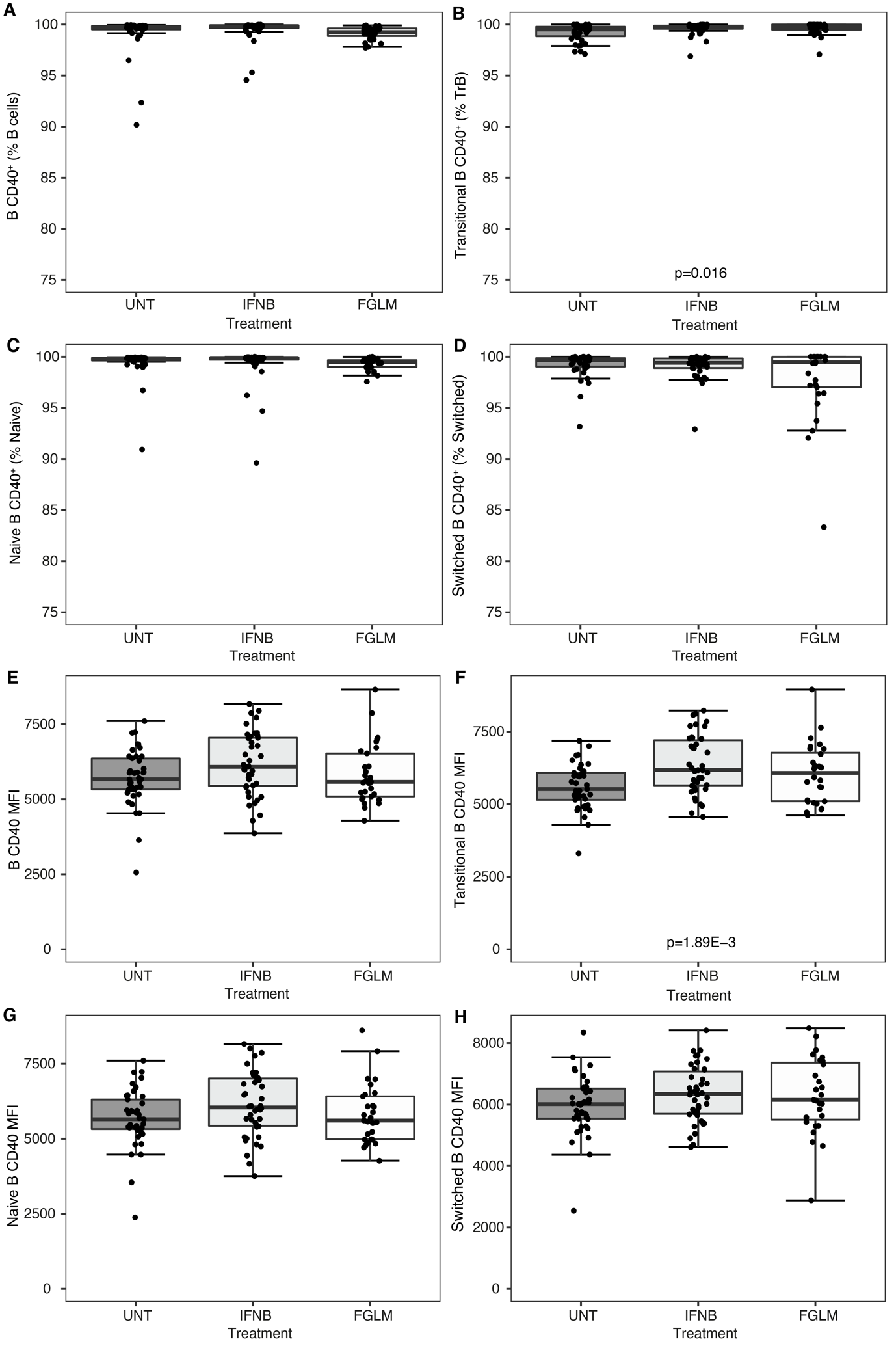


Supplementary Figure 5: Expression levels of BAFF-R on TACI negative and positive B cell subsets according to treatment status.

Association of treatment status with mean fluorescence intensity (MFI) of BAFF-R on **A.** TACI^-^ transitional B, **B.** TACI^+^ transitional B, **C.** TACI^-^ Naive B**, D.** TACI^+^ Naive B, **E.** TACI^-^ switched B cells, **F.** TACI^+^ switched B cells (N_UNT_ = 41, N_IFNB_ = 42, N_FGLM_ = 29). P values ≤ 0.05 are depicted. P values were calculated with a linear regression of the immunological variable in function of treatment status with age and gender as a covariate. We measured expression of BAFF-R and TACI as mean of fluorescence intensity (MFI) across positive cells for each cell type. Box-whisker plots represent median, quartiles and 1.5 x IQR.


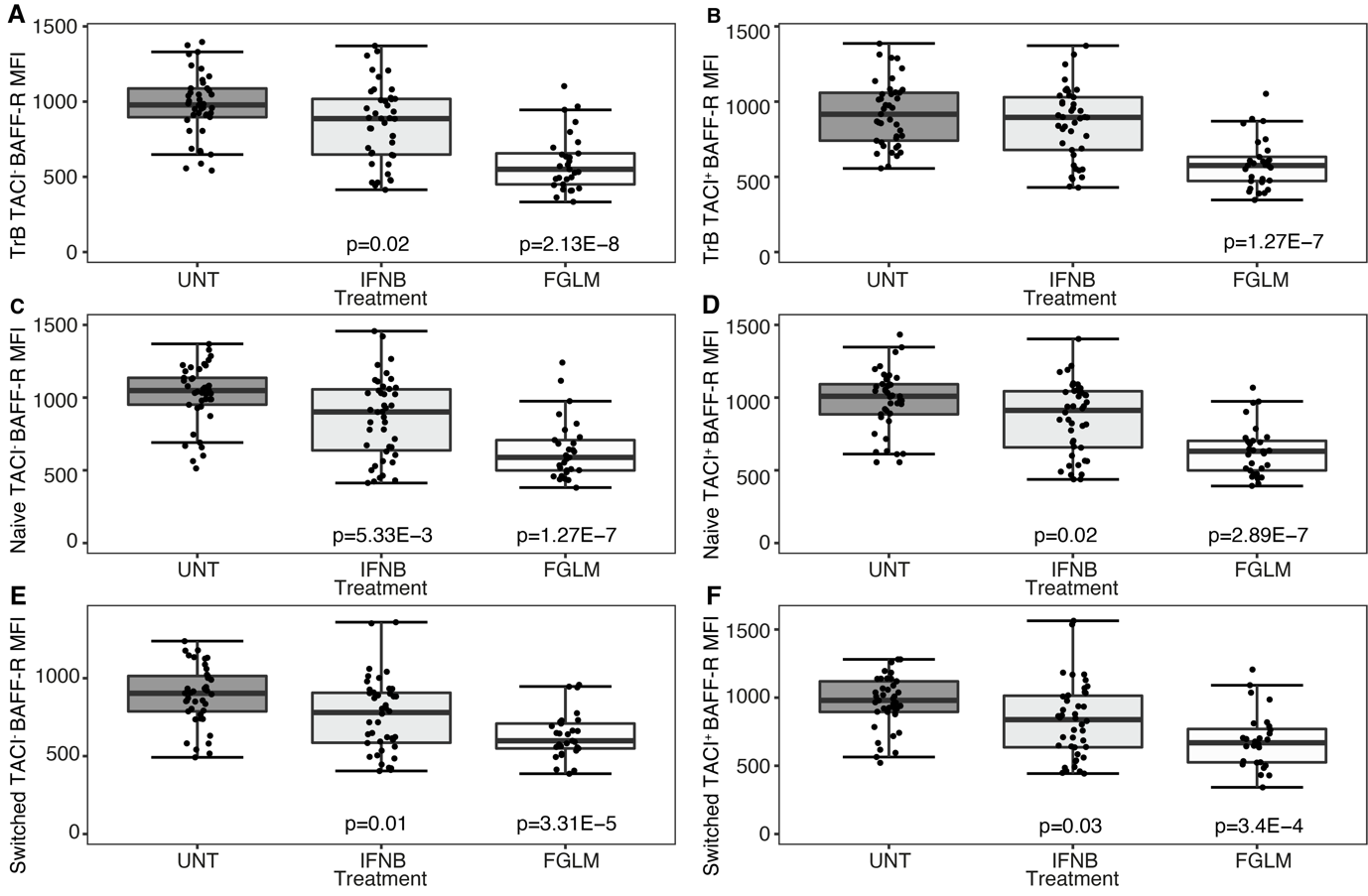

Supplement: Supplementary file 1 [file DataSheet_1.docx]
